# Peptídeos Natriuréticos e Estresse Cardíaco: Hora de Triar a População Assintomática de Alto Risco para Prevenir Casos de Insuficiência Cardíaca?

**DOI:** 10.36660/abc.20230910

**Published:** 2024-08-14

**Authors:** Humberto Villacorta, Davyson Gerhardt de Souza, Antonio José Lagoeiro Jorge

**Affiliations:** 1 Universidade Federal Fluminense Niterói RJ Brasil Universidade Federal Fluminense, Niterói, RJ – Brasil

**Keywords:** Insuficiência Cardíaca, Peptídeos Natriuréticos, Incidência

A insuficiência cardíaca (IC) é a última fase comum de diversas doenças cardíacas. É altamente predominante, principalmente em idosos, e apresenta elevada taxa de morbidade e mortalidade se não tratada adequadamente.^1 - 3^ Apesar dos avanços no tratamento da IC, o número de pessoas afetadas continua significativo e representa um desafio para o sistema de saúde, tanto mundialmente quanto no Brasil.^4 , 5^ Projeções indicam um aumento no número de casos nas próximas décadas, impulsionado pelo envelhecimento da população e pelo aumento da sobrevida em condições cardiovasculares como síndromes coronarianas agudas, valvopatias, arritmias, cardiopatias congênitas, entre outras.^6^ Portanto, a prevenção da IC é fundamental.

Os peptídeos natriuréticos – BNP e NT-proBNP – são os biomarcadores padrão ouro para a IC.^1 - 3^ Foram inicialmente utilizados para diagnosticar a IC em pacientes com Dispneia Aguda.^7 - 9^ Logo, foi demonstrado que também eram excelentes marcadores prognósticos em pacientes com IC aguda.^10 , 11^ Seu uso foi expandido, incluindo pacientes ambulatoriais com IC, conforme recomendado pelas principais diretrizes, para descartar IC ambulatorial em pacientes com sintomas sugestivos e também como marcador prognóstico.^1 - 3^

Indivíduos de alto risco, como aqueles com diabetes mellitus ou hipertensão, mesmo assintomáticos, podem estar predispostos a desenvolver eventos cardiovasculares, incluindo IC incidente. Nesta fase inicial, o dano miocárdico causado por esses fatores de risco é assintomático, não sendo detectado por exames de imagem, um estado conhecido como estresse cardíaco. No entanto, níveis elevados de peptídeos natriuréticos podem indicar a presença de estresse cardíaco.^12^

Um dos primeiros estudos que abordaram essa questão foi realizado com a população descendente de Framingham.^13^ Neste estudo de base populacional, o BNP foi medido no início do estudo em 3.346 indivíduos sem IC, que foram acompanhados por aproximadamente cinco anos. O BNP basal demonstrou ser um preditor independente de eventos cardiovasculares, como morte, primeiro evento cardiovascular significativo, fibrilação atrial, acidente vascular encefálico ou ataque isquêmico transitório e incidência de IC. É importante ressaltar que os pontos de corte derivados deste estudo para predição de risco foram significativamente inferiores aos estabelecidos para o diagnóstico de IC, sendo 20 pg/mL para homens e 23 pg/mL para mulheres.

Nosso grupo publicou recentemente um estudo semelhante, no qual o BNP foi medido no início do estudo em 560 indivíduos, selecionados aleatoriamente em um sistema de atenção primária, que foram acompanhados por cinco anos.^14^ O BNP foi um indicador independente de óbitos, por todas as causas ou hospitalização cardiovascular em pacientes com e sem IC. Embora não tenhamos excluído pacientes com IC no início do estudo, destacamos que 88,6% estavam livres de IC no momento da inclusão.

Em populações de alto risco, como pacientes com diabetes mellitus, foi demonstrado que o NT-proBNP é um indicador de eventos cardiovasculares.^15 - 17^ No estudo de Malachias et al., o NT-proBNP foi o maior indicador de óbito e eventos cardiovasculares e, por si só, demonstrou poder discriminatório semelhante a um modelo formado por 20 variáveis clínicas importantes.^15^

Alguns estudos sugerem que os peptídeos natriuréticos podem identificar indivíduos de alto risco que se beneficiam de acompanhamento e tratamento especializado.^18 , 19^ No estudo STOP-HF,^18^ 1.374 indivíduos assintomáticos e com fatores de risco cardiovascular foram acompanhados por aproximadamente quatro anos. Os indivíduos foram divididos em dois grupos: a) grupo de tratamento convencional, realizado pelo médico da atenção primária (677 participantes); b) grupo triado com BNP. Aqueles que apresentavam BNP > 50 pg/mL constituíram o grupo de intervenção (263 participantes), no qual os indivíduos foram submetidos à ecocardiografia e acompanhados e tratados por um grupo especializado de cuidados cardiovasculares em colaboração com o médico da atenção primária. O grupo de intervenção foi submetido a mais investigações cardiovasculares e recebeu mais tratamento baseado no sistema renina-angiotensina-aldosterona. O grupo intervenção apresentou menor incidência de disfunção sistólica do ventrículo esquerdo, com ou sem IC (5,3% vs 8,7%; com *odds ratio* [OR] de 0,55, intervalo de confiança de 95% 0,37-0,88, p=0,01) e uma menor incidência de IC (1% vs 2,1%; OR de 0,48, intervalo de confiança de 95% 0,20-1,20, p=0,12). Além disso, apresentaram taxas de hospitalização menores (22,3% por 1.000 pacientes/ano vs 40,4%; taxa de incidentes 0,60, intervalo de confiança de 95% 0,45-0,81, p=0,002).

No estudo PONTIAC,^19^ 300 indivíduos com diabetes mellitus, sem cardiopatia e com NT-proBNP > 125 pg/mL foram randomizados para tratamento convencional, realizado em clínicas de diabetes, ou para tratamento intensivo, realizado por meio de acompanhamento adicional com cardiologistas, que realizaram titulações dos inibidores da enzima conversora de angiotensina e dos betabloqueadores. O grupo de tratamento intensivo apresentou redução de 65% na mortalidade cardíaca ou nas taxas de hospitalização em comparação ao grupo convencional, ao longo de um período de acompanhamento de dois anos. O estudo em andamento PONTIAC II^20^ teve como objetivo confirmar este achado em uma população maior.

Com base nas conclusões dos estudos STOP-HF^18^ e PONTIAC,^19^ a Diretriz da ACC/AHA/HFSA de 2022 para o controle da IC apresenta uma recomendação de classe IIa, nível de evidência B, para triagem de indivíduos em risco de desenvolver IC com BNP ou NT-proBNP. Eles afirmam que esta estratégia pode ser útil para a prevenção do desenvolvimento de disfunção do ventrículo esquerdo ou de novo início de IC.^2^

**Figura 1:**
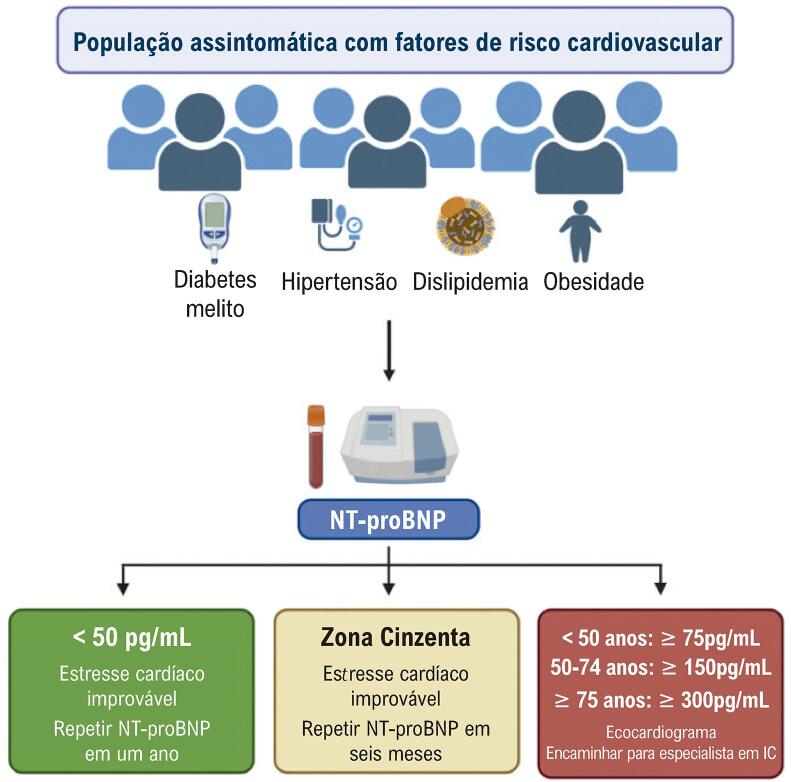
– *Proposta de algoritmo para detecção de estresse cardíaco em indivíduos com fatores de risco cardiovascular na atenção primária. Os pontos de corte de NT-proBNP são os sugeridos pela Associação Insuficiência Cardíaca da Sociedade Europeia de Cardiologia. Os pontos de corte na caixa vermelha à direita são ajustados pela idade. Modificado a partir da referência 12. IC: insuficiência cardíaca; a: anos de idade.*

A Associação de Insuficiência Cardíaca (Heart Failure Association - HFA) da Sociedade Europeia de Cardiologia (European Society of Cardiology - ESC) publicou recentemente um Consenso no qual sugere pontos de corte específicos para NT-proBNP em diferentes cenários.^12^ Com foco no NT-proBNP, uma vez que este é o peptídeo mais utilizado para o controle da IC na Europa, não sendo afetado por medicamentos que atuam na degradação do BNP, como o Sacubitril/Valsartan. O Consenso recomenda o uso do NT-proBNP em pacientes assintomáticos, sem IC estabelecida, na presença de fatores de risco cardiovascular. O documento reconhece a importância desta situação clínica na prevenção da IC e de eventos cardiovasculares e sugere a denominação “estresse cardíaco” para categorizar esta população. A Figura 1 demonstra um algoritmo prático sugerido para o diagnóstico e controle do estresse cardíaco. Em indivíduos com valores de NT-proBNP abaixo do ponto de corte de 50 pg/mL, o diagnóstico de estresse cardíaco é muito improvável, e eles podem ser acompanhados pelo médico de atenção primária, sem investigação adicional. Neste caso, o NT-proBNP deverá ser repetido em um ano. Pacientes acima dos pontos de corte estratificados por idade devem ser submetidos a ecocardiografia e avaliação por um especialista em IC. Entre esses dois grupos, existe uma faixa com valores intermediários de NT-proBNP, denominada zona cinzenta. Nesta faixa, o estresse cardíaco é improvável e o NT-proBNP deve ser repetido em seis meses.

Embora esses pontos de corte ainda precisem de validações prospectivas, concordamos plenamente com o algoritmo HFA-ESC. Estudos anteriores utilizaram um único ponto de corte de NT-proBNP para diagnóstico de estresse cardíaco (> 125 pg/mL).^19^ Um ponto de corte de exclusão mais baixo, conforme sugerido pelo consenso HFA-ESC (< 50 pg/mL), provavelmente aumentará a sensibilidade, sendo mais apropriado para indivíduos assintomáticos. Por outro lado, a introdução de pontos de corte de diagnóstico, estratificados por idade, evita ecocardiogramas e encaminhamentos desnecessários. Além disso, os pontos de corte estratificados por idade são importantes porque corrigem fatores que aumentam os níveis de NT-proBNP, como disfunção renal e fibrilação atrial, mais comuns em idosos. Um ponto de corte de exclusão para BNP provavelmente seria em torno de 20 pg/mL com base no Estudo Framingham Offspring, mas nenhum ponto de corte de diagnóstico foi sugerido.^13^

Em resumo, a IC representa um fardo para o sistema de saúde e todos os esforços devem ser envidados na prevenção de casos incidentes. Os peptídeos natriuréticos são úteis como ferramentas de triagem para identificar indivíduos com risco de desenvolver IC, mas é necessário validar prospectivamente os pontos de corte sugeridos. Consideramos que este é o momento para iniciarmos o rastreamento da população assintomática de alto risco para prevenir a ocorrência de IC.
