# Supplementary material for: Peptídeos Natriuréticos e Estresse Cardíaco: Hora de Triar a População Assintomática de Alto Risco para Prevenir Casos de Insuficiência Cardíaca?
Source: Arq Bras Cardiol. 2024 Aug 14;121(8):e20230910. [Article in Portuguese] doi: 10.36660/abc.20230910 (PMC12092041; doi:10.36660/abc.20230910)
Supplement: Translation

# Translation: Natriuretic Peptides and Heart Stress: Time to Screen the Asymptomatic High-Risk Population to Prevent Incident Heart Failure?

**DOI:** 10.36660/abc.20230910i

**Authors:** Humberto Villacorta, Davyson Gerhardt de Souza, Antonio José Lagoeiro Jorge

**Affiliations:** 1 Brazil Universidade Federal Fluminense, Niterói, RJ – Brazil

**Keywords:** Heart Failure, Natriuretic Peptides, Incidence

Heart failure (HF) is the final common pathway of many cardiac disorders. It is highly prevalent, especially in the elderly, and has high morbidity and mortality if not treated properly.^1 - 3^Although the treatment of HF has evolved, the number of affected individuals is still large and represents a burden on the healthcare system, globally and also in Brazil.^4 , 5^The number of cases is expected to increase over the coming decades, due to the aging of the population and increased survival from cardiovascular conditions, such as acute coronary syndromes, valvular heart diseases, arrhythmias, congenital heart diseases, among others.^6^Therefore, it is imperative to prevent HF.

Natriuretic peptides – BNP and NT-proBNP – are the gold standard biomarkers in HF.^1 - 3^It was used to diagnose HF in patients with Acute Dyspnea.^7 - 9^It was soon demonstrated that they were also excellent prognostic markers in patients with acute HF.^10 , 11^Its use was then expanded to outpatients with HF, being recommended in the main guidelines for ruling out ambulatory HF in patients with symptoms suggestive of HF and also as a prognostic marker.^1 - 3^

High-risk individuals, such as those with diabetes mellitus or hypertension, although asymptomatic may be at risk of developing cardiovascular events, including incident HF. In this initial phase, the damage to the myocardium caused by these risk factors is asymptomatic and not detected by imaging tests, a situation called heart stress. However, the heart stress may be detected by higher levels of natriuretic peptides.^12^

One of the first studies addressing this issue was a study with the Framingham offspring population.^13^In this population-based study, BNP was measured at baseline in 3,346 individuals without HF, who were followed for approximately five years. Baseline BNP was an independent predictor of cardiovascular events, such as death, first major cardiovascular event, atrial fibrillation, stroke or transient ischemic attack, and incidence of HF. It is important to highlight that the cutoffs derived from this study for risk prediction were much lower than those established for the diagnosis of HF, being 20 pg/mL for men and 23 pg/mL for women.

Our group recently published a similar study, in which BNP was measured at baseline in 560 individuals randomly selected from a primary care system, who were followed for five years.^14^BNP was an independent predictor of death from all causes or cardiovascular hospitalization in patients with and without HF. Although we did not exclude patients with HF at baseline, we emphasize that 88.6% were free of HF at the moment of inclusion.

In high-risk populations, such as patients with diabetes mellitus, NT-proBNP has been shown to predict cardiovascular events.^15 - 17^In the study by Malachias et al., NT-proBNP was the major predictor of death and cardiovascular events and, by itself, demonstrated a discriminatory power similar to a model formed by 20 important clinical variables.^15^

Some studies suggest that natriuretic peptides can identify high-risk individuals who benefit from specialized monitoring and treatment.^18 , 19^In the STOP-HF study,^18^1,374 asymptomatic individuals with cardiovascular risk factors were monitored for approximately four years. They were divided into two groups: a) conventional treatment group, carried out by the primary care physician (677 participants); b) group screened with BNP. Those who had BNP > 50 pg/mL constituted the intervention group (263 participants), where the individuals underwent echocardiography and were monitored and treated by a specialized cardiovascular care group in collaboration with the primary care physician. The intervention group underwent more cardiovascular investigations and received more renin-angiotensin-aldosterone system-based treatment. The intervention group had a lower incidence of Left Ventricle (LV) systolic dysfunction, with or without HF (5.3% vs 8.7%; odds ratio [OR] 0.55, 95% confidence interval [CI] 0.37-0.88, p=0.01) and a lower incidence of HF (1% vs 2.1%; OR 0.48, 95% CI 0.20-1.20, p = 0.12). Furthermore, they had lower hospitalization rates (22.3% per 1,000 patients/year vs 40.4%; incident rate ratio 0.60, 95% CI 0.45-0.81, p = 0.002).

In the PONTIAC study,^19^300 individuals with diabetes mellitus, without heart disease, and with NT-proBNP > 125 pg/mL were randomized to conventional treatment, carried out in diabetes clinics, or to intensive treatment, carried out through additional monitoring with cardiologists, who performed the Angiotensin-Converting Enzyme inhibitors and beta blocker titrations. The intensive treatment group showed a 65% reduction in cardiac mortality or hospitalization rates compared to the conventional group, over a two-year follow-up. The ongoing PONTIAC II study^20^was designed to confirm this finding in a larger population.

Based on the findings of the STOP-HF^18^and PONTIAC^19^studies, the 2022 ACC/AHA/HFSA Guideline for the management of HF provides a class IIa recommendation, level of evidence B, for screening individuals at risk of developing HF with BNP or NT-proBNP. They state that this strategy can be useful to prevent the development of LV dysfunction or new onset HF.^2^

The Heart Failure Association (HFA) of the European Society of Cardiology (ESC) recently published a Consensus in which it suggests specific cutoffs for NT-proBNP in different scenarios.^12^They focused on NT-proBNP because it is the most utilized peptide for HF treatment in Europe, since it is not affected by medications that act on the degradation of BNP, such as Sacubitril/Valsartan. The Consensus recommends the use of NT-proBNP in asymptomatic patients, without established HF, in the presence of cardiovascular risk factors. The document recognizes the importance of this clinical situation in preventing HF and cardiovascular events and suggests the name “heart stress” to categorize this population. Figure 1 demonstrates a practical algorithm suggested for the diagnosis and management of heart stress. In individuals with NT-proBNP values below the cutpoint of 50 pg/mL, the diagnosis of heart stress is very unlikely and they can be followed by the primary care physician, with no further investigation. In this case, NT-proBNP should be repeated in one year. Patients above the age-stratified cutpoints should undergo echocardiography and assessment by a HF specialist. Between these two groups, there is a range with intermediate values of NT-proBNP, called the grey zone. In this range, heart stress is unlikely, and NT-proBNP should be repeated in six months.

**Figure 1:** – *Proposed algorithm for the detection of heart stress in individuals with cardiovascular risk factors in primary care. NT-proBNP cutpoints are those suggested by the Heart Failure Association of the European Society of Cardiology. Cutoffs in the red box on the right are age-adjusted. Modified from reference 12. HF: heart failure; y: years of age.*

Although these cutpoints still need prospective validations, we very much agree with the HFA-ESC algorithm. Previous studies used a single NT-proBNP cutoff for diagnosing heart stress (> 125 pg/mL).^19^A lower rule-out cutoff as suggested by the HFA-ESC consensus (< 50 pg/mL) will likely increase the sensitivity and is more appropriate for asymptomatic individuals. On the other hand, the introduction of age-stratified rule-in cutoffs avoids unnecessary echocardiograms and referrals. Additionally, age-stratified cutoffs are important as they correct for factors that increase NT-proBNP levels, such as renal dysfunction and atrial fibrillation, which are more common in the elderly. A rule-out cutoff for BNP would likely be around 20 pg/mL based on the Framingham Offspring Study but no rule-in cutoffs have been suggested.^13^

In summary, HF is a burden to the health system and all efforts should be used in the prevention of incident cases. Natriuretic peptides are useful tools for screening individuals at risk of developing HF, although we need prospective validation of the suggested cutpoints. We believe the time has come to screen the asymptomatic high-risk population and thus prevent incident HF.
